# Extreme Fuzziness: Direct Interactions between Two IDPs

**DOI:** 10.3390/biom9030081

**Published:** 2019-02-26

**Authors:** Wenning Wang, Dongdong Wang

**Affiliations:** Department of Chemistry, Institute of Biomedical Sciences and Multiscale Research Institute of Complex Systems, Fudan University, Shanghai 200438, China; 14110220035@fudan.edu.cn

**Keywords:** intrinsic disordered protein, extremely fuzzy complex, protein interaction, binding mechanism

## Abstract

Protein interactions involving intrinsically disordered proteins (IDPs) greatly extend the range of binding mechanisms available to proteins. In interactions employing coupled folding and binding, IDPs undergo disorder-to-order transitions to form a complex with a well-defined structure. In many other cases, IDPs retain structural plasticity in the final complexes, which have been defined as the fuzzy complexes. While a large number of fuzzy complexes have been characterized with variety of fuzzy patterns, many of the interactions are between an IDP and a structured protein. Thus, whether two IDPs can interact directly to form a fuzzy complex without disorder-to-order transition remains an open question. Recently, two studies of interactions between IDPs (4.1G-CTD/NuMA and H1/ProTα) have found a definite answer to this question. Detailed characterizations combined with nuclear magnetic resonance (NMR), single-molecule Förster resonance energy transfer (smFRET) and molecular dynamics (MD) simulation demonstrate that direct interactions between these two pairs of IDPs do form fuzzy complexes while retaining the conformational dynamics of the isolated proteins, which we name as the extremely fuzzy complexes. Extreme fuzziness completes the full spectrum of protein-protein interaction modes, suggesting that a more generalized model beyond existing binding mechanisms is required. Previous models of protein interaction could be applicable to some aspects of the extremely fuzzy interactions, but in more general sense, the distinction between native and nonnative contacts, which was used to understand protein folding and binding, becomes obscure. Exploring the phenomenon of extreme fuzziness may shed new light on molecular recognition and drug design.

## 1. Introduction

A stable three-dimensional structure of a protein is the key to understanding protein function in the conventional structure-function paradigm [1]. On the other hand, it has long been recognized that proteins are ‘soft matter’ whose conformational fluctuation has functional significance [2]. Generally speaking, proteins are dynamic across a wide range of time scales and amplitudes, from pico-second bond vibrations to second-minute folding process. For a well-structured protein, the conformational dynamics extensively studied include fast motion of side chains, flexibility of local segments (such as loops) or collective motions sampling the conformational space around the stable (native) structure [3]. By incorporating conformational dynamics, the ‘structure-function’ paradigm expands to a ‘structure-dynamics-function’ one [3,4,5]. Since two decades ago, this framework has been challenged by the recognition of the considerable amount and intriguing characteristics of proteins without well-folded structures, i.e., intrinsically disordered proteins (IDPs) [6,7,8,9,10,11].

Intrinsically disordered proteins do not have a unique stable structure, or a unique folding funnel on the free energy landscape [12]. Intrinsically disordered proteins or intrinsically disordered domains/regions (IDRs) are abundant in all organisms, especially in eukaryotic proteomes. In the human genome, around 40% of protein-coding genes contain disordered regions of >30 amino acids in length [13,14,15]. Nevertheless, IDPs have been ‘dark matter’ in structural biology for a long time because they are difficult to characterize with traditional tools of biophysics. Today, increasing evidence has revealed that IDPs are implicated in various important biological functions, including signal transduction, regulation, gene transcription and replication. Probably due to the structural disorder, the predominant function of IDPs is protein-protein interaction although enzyme activity of IDP was discovered recently [16].

Understanding protein-protein interaction involving IDPs is a central theme for experimental and theoretical studies of IDPs [17]. Our knowledge about it has gone through several stages [18]. The early established prototype of IDP-mediated interaction is the so-called folding upon binding, or coupled folding and binding, in which the IDP folds into well-defined structure upon complex formation [19]. On the other hand, structural heterogeneity and flexibility have been found in many protein complexes involving IDPs. In one scenario referred to as polymorphism, the disordered protein/peptide folds into stable structure but forms alternative conformations in the final complex [20]. In other cases, however, part of the IDP retains structural flexibility, experiencing rapid conformational exchange [21,22,23]. In yet another situation, several binding sites on the IDP compete to bind a single site on the receptor, and the binding could be described as rapid switching among the interactions of the receptor with alternative sites on the IDP [24]. To describe all these binding modes other than the traditional interactions between structured proteins, Tompa and Fuxreiter proposed the notion of fuzziness and fuzzy complex [25]. Initially, the term ‘fuzzy complex’ was used to refer to all kinds of protein complexes involving structural heterogeneity and flexibility, which encompasses a broad spectrum of protein interactions mediated by IDPs [25]. Fuzziness also occurs in intramolecular interactions, functioning as a signal sensor [26]. Recently, Olsen et al., proposed that fuzziness should have a more strict definition, and they define it as ‘two or more ligand binding sites on the receptor being able to bind to two or more receptor binding sites on the ligand’ [27]. In other words, multivalency plays a central role in fuzzy interactions [28]. According to this definition, the binding interface remains highly dynamic in the complex, and the examples mentioned above do not belong to fuzzy complexes. For example, two complexes can be categorized to such strictly defined fuzzy complexes, which are complex between nucleoporins and nuclear transporter receptors [29,30], and complex between clathrin heavy chain and assembly protein 180 kDa (AP180) [31,32,33]. It is noticeable that almost all these IDP mediated protein interactions involve one well-structured protein. Early studies of the dimerization of the intracellular region of the T cell receptor subunit ζ had provided indications of fuzzy complex formation between IDPs [34,35], but it was questioned by later experimental evidence [36]. Therefore, whether two IDPs can interact directly to form a fuzzy complex while retaining structural plasticity remains an open question [37]. Recently, this question has been answered by two studies [38,39] with detailed characterizations of the interactions between two pairs of IDPs that form dynamic fuzzy complexes. In both cases, the structural disorder and conformational dynamics of the two interacting IDPs are preserved in the complexes. To distinguish this type of fuzzy complexes from those discovered before, we call them the extremely fuzzy complexes.

## 2. Interaction between 4.1G-CTD and NuMA

**Figure 1:**
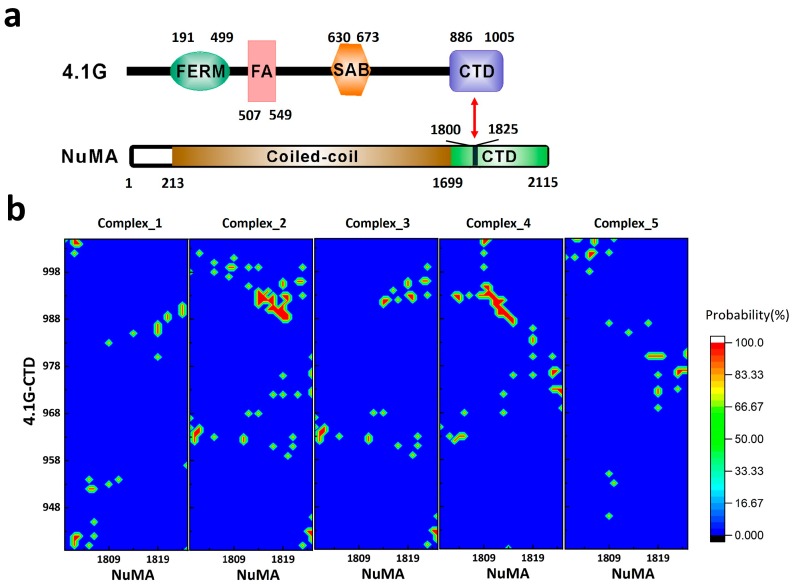
(**a**) The domain organization of 4.1G and nuclear mitotic apparatus (NuMA). (**b**) The contact maps between 4.1G-C-terminal domain (CTD) and NuMA in the top five clusters of 4.1G-CTD/NuMA structure ensemble based on replica exchange molecular dynamics (REMD) simulations in [38]. FERM: four.one–ezrin–radixin–moesin domain; FA: FERM adjacent domain; SAB: spectrin–actin binding domain.

**Figure 2:**
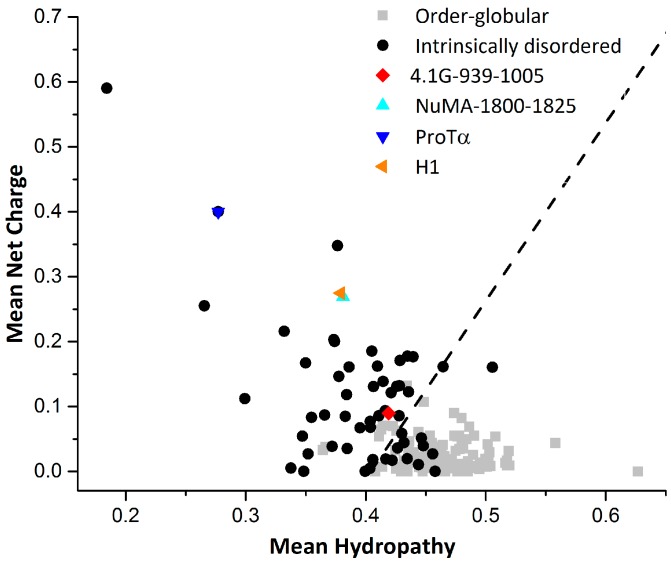
Charge hydropathy ratio for proteins. The dotted line represents an empirically determined charge/hydropathy relationship that distinguishes most ordered globular and intrinsically disordered proteins. The ratio was calculated using the Predictor of Natural Disordered Regions (PONDR) and the data of ordered proteins and disordered proteins were taken from PONDR website [48].

The first clearly characterized extremely fuzzy complex is 4.1G-CTD/NuMA [38]. Protein 4.1 is a ubiquitously expressed adaptor protein, which serves as a hub organizing signaling complexes involving many membrane proteins [40]. All members in protein 4.1 family (4.1R, 4.1G, 4.1N, and 4.1B) have two common functional domains: a four.one–ezrin–radixin–moesin (FERM) domain and a C-terminal domain (CTD) (Figure 1a) [40]. It was recently discovered that the interaction of 4.1G/4.1R-CTD with the nuclear mitotic apparatus (NuMA) protein plays a key role in NuMA localization during symmetric [41] and asymmetric [42] cell divisions. The C-terminal region of NuMA that interacts with 4.1G is a 26-amino acid disordered fragment (Figure 1a), while NMR shows that 4.1G-CTD is also intrinsically disordered (Figure 2a in [38]). Interestingly, the specific interaction between the two proteins does not induce structure formation in the complex. Titration of NuMA induces resonance line broadening on the heteronuclear single quantum coherence (HSQC) spectrum of 4.1G-CTD, but the chemical shift dispersion remained limited without obvious chemical shift changes (Figure S5 in [38]). Single-molecule Förster resonance energy transfer (smFRET) measurements also show that 4.1G-CTD exhibits similar stochastic conformational fluctuations in the free form and in the complex (Figure 5 in [38]). Atomic molecular dynamics (MD) simulations provide great details of the interaction between the two proteins. In contrast to the fuzzy binding between an IDP and a structured protein, the interaction between 4.1G-CTD and NuMA encompasses many contact spots on both 4.1G and NuMA without a fixed binding interface. Nevertheless, the binding sites could be clearly identified according to the statistics of the contact frequency from the MD simulation trajectories (Figure 3b in [38]). Several contact ‘hot spots’ on 4.1G-CTD and NuMA have been verified by point mutagenesis experiments (Figure 3c in [38]). Therefore, the binding could be described as dynamic and stochastic interactions between multiple sites on both proteins, conforming to the strict definition of fuzziness [27]. Moreover, the binding modulates the structures of both 4.1G-CTD and NuMA. Both smFRET measurement and MD simulation show that the conformational ensemble of 4.1G-CTD was changed by NuMA binding. 4.1G-CTD is basically a molten globule and conformations with similar topological fold have been identified in the free form 4.1G-CTD and complex ensembles (Figures 2d and 3a in [38]). However, the interaction obviously induced local structural changes in both 4.1G-CTD and NuMA, which was reflected in the changes of the secondary structural contents of both proteins in MD simulations. 4.1G-CTD and NuMA experience mutual structural adaptations upon binding. Unlike the case of coupled folding and binding, this adaptation does not lead to a unique and stable structure of complex, but a new conformational ensemble of complex (Figure 3a in [38]).

## 3. Interaction between ProTα and H1

Another clearly characterized extremely fuzzy complex is H1 chaperone/prothymosin-α (H1/ProTα) [39]. Human linker histone H1 is positively charged and largely unstructured. H1 chaperone/prothymosin-α is a completely unstructured protein with high content of negative charges. It has been shown that the two proteins will form a highly dynamic fuzzy complex with extremely high binding affinity (Figure 2c in [39]), although a more recent study gave a much lower binding affinity (Figure 1 in [43]). Nuclear magnetic resonance and circular dichroism (CD) experiments demonstrate that the interaction does not induce any structure formation, neither locally nor globally (Figure 1 in [39]). Single-molecule FRET combined with fluorescence correlation spectroscopy measurements have shown that the long-range distance dynamics in isolated ProTα and H1 are retained in the complex. Interestingly, the different time-scales of the dynamics (chain reconfiguration measured by fluorescence correlation spectroscopy (FCS) in isolated proteins are similar in the complex, indicating the coupling of the dynamics upon binding. Using restraints derived from the experiments and a coarse-grained force field, a structural ensemble of the H1/ProTα complex was constructed through simulation. The intra and intermolecular distance maps indicate that the interactions between ProTα and H1 are broadly distributed along their sequences. In other words, the binding interface is so large that there is barely any specific binding site on both proteins. This is different from the case of 4.1G/NuMA, where frequent contact sites on both proteins could be identified and verified by mutagenesis experiments [38]. Another feature of H1/ProTα complex that differs from 4.1G/NuMA is that the structure ensemble does not show distinct conformational clusters. This is typical for IDPs with highly disordered conformations that resemble statistical coils [13]. The charge/hydropathy (C/H) ratios [44] of ProTα and H1 indicate that the two proteins are IDPs more coil-like (or intrinsic coils [6]) in the two dimensional charge/hydropathy space (Figure 2). The C/H ratio of NuMA is very similar with that of H1, while the C/H ratio of 4.1G-CTD indicates that 4.1G-CTD is more like an intrinsic premolten globule [6] (Figure 2). In line with this conclusion, the structure ensemble of 4.1G-CTD/NuMA exhibits distinct conformational clusters and discrete binding sites [38]. Due to the high content of charged residues, the electrostatic interactions play a major part in the H1/ProTα complex formation. For 4.1G-CTD and NuMA, the calculated binding energy components using the molecular mechanics Poisson-Boltzmann surface area method (MM-PBSA) (Table 1) show that the energy of electrostatic interactions (ΔE_ele_) and the electrostatic contribution to the solvation free energy (ΔG_polar_) are both large in magnitude. On the other hand, the magnitudes of van der Waals interaction (ΔE_vdW_) and nonelectrostatic contributions to solvation free energy (ΔG_nonpolar_) are relatively moderate. The summation of ΔE_ele_ and ΔG_polar_ is positive, i.e., unfavorable for binding, while the nonelectrostatic contributions are all negative. This rough estimation suggests that the binding of 4.1G-CTD to NuMA is not mainly driven by electrostatic interactions and nonpolar interactions have important contribute. This is consistent with the mutagenesis experiments in [38], where mutations of both charged and hydrophobic residues impaired the binding.

So far, 4.1G-CTD/NuMA and H1/ProTα are the only two clearly characterized extremely fuzzy complexes at high resolution, i.e., residue specific and/or atomic level information have been obtained. However, new evidence of extremely fuzzy complexes between two or more IDPs is emerging. For example, evidence for formation of extremely fuzzy complex between human α, β and γ synuclein have been recently reported [46,47].

## 4. How Unique are Extremely Fuzzy Complexes?

The thermodynamics and kinetics of protein-protein association are far more complicated than those of small molecules [49,50,51]. The possible sources of the complication include the relatively weak interaction between proteins, the hydrophobic effect of water, the structural plasticity of polypeptides and the interplays among these [52]. For IDPs, these features are more prominent than structured proteins [37]. The understanding of IDP interactions is built up based on the studies of structured proteins [53,54,55]. Although the underlying physical principles may not be fundamentally different, the detailed mechanistic picture of IDP interaction is definitely more complicated [27,53]. So far, we do not have a clear picture of the recognition mechanism between two IDPs that form an extremely fuzzy complex [27], which may represent the complicated situation in IDP-mediated interactions. In the unbound state, both binding partners have broad conformational distributions. On each IDP, there exist multiple binding sites that could interact with multiple sites on the opposite IDP. At any given time, the two IDPs may interact through one site (monovalent), or through multiple sites simultaneously (multivalent), and the sites on two IDPs may not pair with each other in a unique way. Therefore, the number of the possible combinations of the two sets of binding sites can be quite considerable, corresponding to many different binding interfaces and an ensemble of complexes. This scenario has been pictured in the structure ensemble of 4.1G-CTD/NuMA complex derived from all-atom MD simulations [38]. Both 4.1G-CTD and NuMA have different conformations in various clusters of complexes, where variable binding modes and binding interfaces are adopted. To show this, we calculated the individual contact maps of each representative structure of the top five clusters in the ensemble of 4.1G-CTD/NuMA complex. As presented in Figure 1b, the binding patterns are all different in these five structures, including multiple binding sites and not limited to a single way. In coupled folding and binding model, there is a clear distinction between native and nonnative interactions, which are defined respectively as the interactions included and not included in the final folded complex structure. Deciphering their roles during protein recognition is crucial for understanding the binding mechanism [37]. In the case of extremely fuzzy interaction, however, the distinction between native and nonnative interactions could be obscure. The native interactions can be defined as those highly populated in the structure ensemble of the extremely fuzzy complex. However, compared with the coupled folding and binding cases, the identification of native and nonnative interactions in extremely fuzzy complexes is technically demanding. Generally, it is difficult to obtain an accurate structure ensemble of the fuzzy complex. In the case of 4.1G-CTD/NuMA, the residue pairs that show high contact probabilities (Figure 1b in this paper and Figure 3b in [38]) could be defined as native interactions, while those with negligible probabilities are nonnative. However, when inter-residue contact probabilities distribute more evenly along the sequence of the two proteins, the distinction between native and nonnative interactions is less obvious. For example, in the complex of H1/ProTα, the two IDPs seem to have a greatly extended binding interface, and the structure model derived from simulation demonstrates that almost all amino acids in the two proteins are in close contact with their binding partners and the binding is promiscuous at the same time (Figure 4b,c in [39]). In line with this picture, the association rate constant *k*_on_ of H1/Pro-Tα interaction is at the diffusion limit (3.1 ± 0.1 × 10^9^ M^−1^ s^−1^), suggesting that the binding process is basically barrierless. It is anticipated that for extremely fuzzy complexes similar to H1/Pro-Tα, i.e., with extended conformations and very broad binding interface, the association is basically diffusion limited.

Although the binding mechanism of extremely fuzzy complex lacks a simple picture, some established explanations for other types of IDP-mediated interactions with different degrees of fuzziness could be applicable in certain aspect. In coupled folding and binding model, it has been proposed that structure element similar to those in complex are preformed in unbound IDPs [56] and the binding process follows conformational selection mechanism. In extremely fuzzy interactions, both free form proteins and complex have broad conformational distributions. Therefore, many structures or structure elements in extremely fuzzy complex are already present in the unbound structure ensemble. The binding process could be roughly described by population shift of the structure ensemble. As in the case of 4.1G-CTD/NuMA complex, some secondary structures in isolated 4.1G-CTD are retained in the complex, and the tertiary folds of free form 4.1G-CTD does not dramatically differ from those in complex (Figures 2d and 3a in [38]). NuMA binding modulates the conformational ensemble of 4.1G-CTD as observed in the smFRET measurement and MD simulation [38]. On the other hand, the two IDPs also experience mutual modulation of their structures during the binding process. Therefore, an induced-fit mechanism is always present in the IDP-IDP interaction.

**Figure 3:**
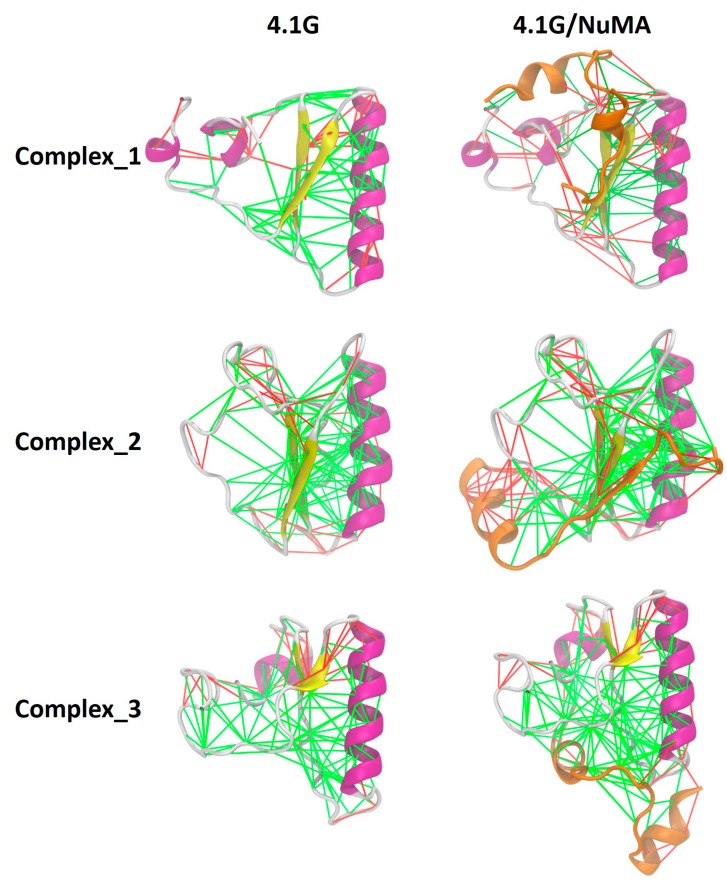
NuMA binding does not reduce the local frustrated interactions in 4.1G-CTD. Frustrations of pair interactions in the top three clusters of 4.1G/NuMA complex are evaluated. The green lines indicate minimally frustrated interactions, while the red lines indicate highly frustrated interactions. Representations in the left column are free form 4.1G-CTD and the ones in the right column are 4.1G/NuMA complex. NuMA peptide is colored orange and 4.1G is colored according to its secondary structure.

From the perspective of energy landscape [4], the binding landscape and the landscape of the final complex are all highly frustrated. Frustration is a well-defined concept in physics, and Frauenfelder et al. introduced it to protein folding theory more than two decades ago [4]. For IDPs, it means there are many local minima separated by low barriers on the energy landscape. Therefore, no single native state dominates for IDPs. For structured proteins, folding is a process with significant minimization of frustration. In the folding upon binding mechanism of IDPs, there is also a remarkable minimization of frustration. In extremely fuzzy complexes such as 4.1G-CTD/NuMA and H1/ProTα, the free energy landscape remains highly frustrated since the complex structure ensemble remains a broad conformational distribution. In addition, the promiscuous binding modes manifested in the complex structure ensembles of both 4.1G-CTD/NuMA and H1/ProTα imply that the binding landscape is also frustrated. Due to these observations, we may speculate that the overall minimization of frustration upon complex formation in extremely fuzzy interactions is limited. To examine the local effects of fuzzy interactions in terms of frustration, we calculated the frustrations of pair interactions in three 4.1G-CTD/NuMA complex structures (representative structures of the top three clusters derived from replica exchange molecular dynamics (REMD) simulations in [38]) by using the “Frustratometer” web server (http://frustratometer.qb.fcen.uba.ar/) [57] As shown in Figure 3, the highly frustrated interactions (red lines) in free form structures of 4.1G-CTD are retained in the complexes, i.e., NuMA binding does not lead to obvious local frustration reduction. This is consistent with the main feature of extremely fuzzy complex.

As mentioned above, it is difficult to define native and nonnative contacts in the binding of two IDPs. This situation is especially obvious in the case of H1/Pro-Tα. As for the 4.1G-CTD/NuMA complex, it might be possible to identify these two contact types since specific binding sites have been found. Thus, even for the two complexes representing the extremely fuzzy interactions, the detailed binding mechanisms could be different. In general, the task of achieving a holistic understanding of the mechanism for IDP-mediated interactions with various degrees of fuzziness will require the identification and evaluation of relative contributions of native and nonnative contacts, which is beyond conventional conformational selection and induced-fit models [37,58,59]. The extremely fuzzy complexes, however, pose additional challenges to this task, suggesting that the categorization of folded and not folded is no longer the essential concern [58], and the discrimination of native and nonnative contacts should be re-evaluated. Such detailed mechanistic characterization of IDPs is challenging for conventional structure biology techniques, and molecular simulation and theory is valuable complement to experiment [60,61,62,63]. On the experimental side, combination of various techniques complementary in time and space resolution is the optimal strategy. On the computational and theoretical side, molecular simulations contribute crucially to the generation of conformational ensembles [58,64]. The reliability of molecular simulation relies on further optimization of force field for disordered proteins and development of enhanced sampling methods [64]. Beyond the equilibrium conformational ensemble, exploring binding mechanism of fuzzy complex using molecular simulation requires unbiased time-evolution trajectories to obtain correct dynamic information, and many enhanced sampling techniques are not applicable. Moreover, based on accumulating data from both experimental and computational studies, development of analytical theory is expected to make testable prediction for experimental investigations of the binding mechanism of fuzzy complexes [27].

The studies of fuzzy complexes in the past two decades have provided hints on our understanding of the extreme fuzziness. The fuzzy complexes database (FuzDB) has collected dozens of fuzzy complexes characterized in detail [65]. The association mechanisms of these fuzzy complexes have been categorized into four classes: (1) conformational selection: the fuzzy regions affect the conformational equilibrium ensemble and promote the formation of secondary structure elements that is compatible for binding; (2) flexibility modulation: the fuzzy regions at the interface participate in the modulation of the binding entropy; (3) tethering: the fuzzy region increases the local concentration of the binding element in the proximity of the partner; (4) competitive binding: intramolecular interactions of the fuzzy region compete with the intermolecular interactions of the binding element [65]. These four categories, obviously, are not mutually exclusive, and many complexes could be categorized into more than one type. Therefore, the concept of fuzziness is more likely a phenomenological description of a wide spectrum of IDP complexes. In practice, the assembly mechanism of fuzzy complexes should be analyzed case by case. Finally, it is worth noting that the functional implication is important for understanding fuzziness in IDP assembly. The organizing principle of IDP fuzzy complexes has been found to manifest its uniqueness through functional roles [18]. For example, the structural heterogeneity and dynamic nature of fuzzy complexes may facilitate interactions with alternative partners simultaneously or consecutively, and posttranslational modification regulated binding etc. Intrinsically disordered proteins and fuzzy interactions are involved in important signaling pathways, and they are also attractive targets for drug design [18,66]. Designing inhibitory small molecules for IDP and dynamic binding requires new strategies, since the target is ensemble rather than a single structure. Conversely, these small molecules could be chemical probes for our understanding of IDP interaction mechanism [66].

## 5. Conclusions

The two extremely fuzzy complexes reported recently have given a definite answer to the question of whether fuzzy complexes can be formed by two IDPs while retaining structural dynamics. This type of fuzziness represents the dynamic extreme in the spectrum of IDP interactions, suggesting that a more general model beyond all previously proposed mechanisms is required to understand protein interactions. In the perspective of energy landscape, the whole pathway of specific protein binding may occur without marked reduction of frustration, and therefore the reconsideration of ‘native’ and ‘nonnative’ contact is necessary. Both mechanistic understanding and functional importance of the extremely fuzzy interactions are exciting aspects in IDP studies.

## Figures and Tables

**Table 1:** Binding energy components of 4.1G-CTD/NuMA obtained from the molecular mechanics Poisson-Boltzmann surface area method (MM-PBSA) calculation using the g_mmpbsa [45] in GROMACS.

	Binding Energy Components (kJ/mol)
ΔE_vdW_	−206.2 ± 2.2
ΔE_ele_	−1496.4 ± 8.9
ΔG_polar_	1653.3 ± 11.9
ΔG_nonpolar_	−36.9 ± 0.2
ΔG_bind_	−86.0 ± 4.7
